# Burnout, Anxiety, Stress, and Depression Among Iranian Nurses: Before and During the First Wave of the COVID-19 Pandemic

**DOI:** 10.3389/fpsyg.2021.789737

**Published:** 2021-11-25

**Authors:** Mohammad Ali Zakeri, Elham Rahiminezhad, Farzaneh Salehi, Hamid Ganjeh, Mahlagha Dehghan

**Affiliations:** ^1^Non-communicable Diseases Research Center, Rafsanjan University of Medical Sciences, Rafsanjan, Iran; ^2^Determinants of Health Research Centre, Rafsanjan University of Medical Sciences, Rafsanjan, Iran; ^3^Student Research Committee, Razi Faculty of Nursing and Midwifery, Kerman University of Medical Sciences, Kerman, Iran; ^4^Nursing Research Center, Kerman University of Medical Sciences, Kerman, Iran; ^5^Clinical Research Center, Rafsanjan University of Medical Sciences, Rafsanjan, Iran

**Keywords:** burnout, anxiety, stress, depression, nurse, COVID-19

## Abstract

**Background:** Nurses are the major healthcare workforce in an epidemic and have the most contact with patients. Frontline nurses face many health challenges during the COVID-19 epidemic, are directly at risk when treating and caring for COVID-19 patients, and thus experience severe stress and problems in the workplace leading to physical, mental, and social disorders, as well as burnout, anxiety, stress, and depression. The purpose of this study was to compare burnout, anxiety, stress, and depression in nurses before and during the first wave of the COVID-19 pandemic.

**Methods:** This was a cross sectional study. We assessed 266 frontline nurses before and 242 frontline nurses during the first wave of the COVID-19 pandemic with one year apart in 2019 and 2020 (two-stage sampling). The data were collected using demographic questionnaire, Maslach Burnout Questionnaire and Depression, Anxiety and Stress Scale (DASS-21) in public hospitals in Southern Iran.

**Results:** There were no significant differences between groups in subscales of burnout (*p* > 0.05). Anxiety, stress and depression scores significantly increased during the first wave of the COVID-19 pandemic compared with before the COVID-19 outbreak (*p* < 0.05). There were significant differences between groups in level of anxiety (*p* < 0.001) and stress (*p* = 0.04). Before the COVID-19 outbreak, burnout predicted 11, 15, and 13% of the variance of anxiety, stress and depression, respectively. In addition, stress, monthly working hours and shift were variables that predicted 16% of the variance of burnout before COVID-19.

**Conclusion:** The results of the present study showed that burnout during the first wave of the COVID-19 pandemic did not change significantly compared with before COVID-19. Anxiety, stress and depression increased significantly first wave of the COVID-19 pandemic.

## Introduction

COVID-19 first appeared in Wuhan, China in December 2019 and quickly spread throughout the world (Chen et al., 2020). On March 11, 2020, the World Health Organization declared the COVID-19 outbreak a pandemic (Sahin et al., 2020). Nurses are the major healthcare workforce in an epidemic (Wan et al., 2020) and have the most contact with patients (Kisa, 2020). Frontline nurses face many health challenges during the COVID-19 epidemic (Zakeri et al., 2021d), are directly at risk when treating and caring for COVID-19 patients, and thus experience severe stress and problems in the workplace leading to physical, mental, and social disorders, as well as burnout (Kisa, 2020), anxiety, stress, and depression (Wan et al., 2020).

Burnout syndrome is a dangerous overwork condition that can lead to physical or mental illness (Guixia and Hui, 2020). This syndrome was first identified in the early 1970s, primarily among health-care workers (Sahin et al., 2020). According to World Health Organization on May 28, 2019, occupational burnout is a syndrome resulting from chronic work-related stress (Wan et al., 2020). Fatigue, general pain, coronary artery disease, respiratory problems, emotional exhaustion, irritability, impaired concentration, depression, decreased self-esteem, and loss of interest in patients are all symptoms of BOS (Kisa, 2020; Sahin et al., 2020). Reduced health-care performance, feeling of negativism related to one’s job, a high rate of turnover in personnel, and increased service costs are symptoms of organizational burnout. With an increasing epidemic, occupational burnout remains a significant risk factor affecting the quality of life and health of healthcare workers, particularly nurses (Hofmeyer et al., 2020).

Wan et al. (2020) investigated factors related to occupational burnout among Chinese nurses during the COVID-19 outbreak. They discovered that nurses had a high level of anxiety and a moderate level of occupational burnout (Wan et al., 2020). Guixia and Hui (2020) discovered a relationship between nurse’s occupational burnout, anxiety, and depression during the COVID-19 epidemic (Guixia and Hui, 2020).

Anxiety is a generalized, unpleasant, and vague feeling of panic with an unknown origin that includes uncertainty, helplessness, and physiological arousal, as well as symptoms such as fatigue, restlessness, and increased heart rate (Vilagut et al., 2016; Liu et al., 2020). Stress is an unavoidable part of everyday life. Work-related stress is defined as emotional, perceptual, behavioral, and physiological reactions to negativism related to one’s job, organization, or workplace (Zakeri et al., 2021a). Depression is one of the most common behavioral disorders, according to the World Health Organization, and is associated with low mood, loss of interest, feelings of guilt and worthlessness, changes in energy, concentration, sleep, and appetite (Vilagut et al., 2016). According to Zakeri et al. (2021a), anxiety, stress, and depression were very common among the frontline workers caring for the COVID-19 patients (Zakeri et al., 2021a). Cai et al. (2020) studied 534 physicians, nurses, and primary care providers in Hubei State and found that they were under a lot of stress during the COVID-19 outbreak (Cai et al., 2020). Anxiety during or after a crisis can impair mental reasoning and abstract thinking skills, as well as cause a lack of attention and coordination in health-care professionals (nurses, physicians, etc.). Anxiety can reduce the effectiveness of healthcare professionals’ efforts to protect people’s health, and direct contact with COVID-19 patients can cause serious concerns like fear of death and virus transmission to family members, as well as feelings of loneliness and anger, all of which can lead to stress and depression (Celmece and Menekay, 2020). When a person is constantly exposed to anxiety and stress, he/she loses confidence and becomes depressed, which increase work-related stress and lower performance. They gradually lose their mental and physical abilities, and eventually develop unstable mental and neurological disorders (Vilagut et al., 2016; Zakeri et al., 2021c). The deadly and uncontrollable nature of COVID-19, together with the relatively high rate of infection and mortality among healthcare professionals, can provoke feelings of anxiety and stress in medical staff. Issues such as social stigmatization, shortage of personal protection equipment supplies, and heavy workload on the staff during the COVID - 19 pandemic can aggravate this situation. Therefore, this pandemic is expected to have a substantial psychological impact on healthcare professionals (Jalili et al., 2021). Studies have revealed the psychological impacts of this life-threating virus on people, especially medical staff. As in Italy, two infected nurses committed suicide due to fear of spreading COVID-19 to patients. It is possible that fear and anxiety of falling sick or dying, and helplessness will drive increased suicide rates in 2020 (Alizadeh et al., 2020).

As a result, these factors may have an impact on nurses’ job performance and health and lower their quality of life (Celmece and Menekay, 2020). Celmece and Menekay (2020) showed the impact of stress, anxiety, and occupational burnout on the quality of life of health care professionals (physicians, nurses, and health care assistants) caring for the COVID-19 patients during pandemic (Celmece and Menekay, 2020).

The physical and mental health of nurses is critical for providing health care during the COVID-19 epidemic. Identifying the factors that contribute to occupational burnout, anxiety, stress, and depression in nurses can thus help develop strategies to address these issues. Furthermore, no study compared burnout, anxiety, stress, and depression in nurses before and during the first wave of the COVID-19 pandemic. As a result, the purpose of this study was to compare occupational burnout, anxiety, stress, and depression in nurses before and during the first wave of the COVID-19 pandemic.

## Materials and Methods

### Study Design and Setting

A cross-sectional study was used to investigate the effect of burnout, anxiety, stress, and depression on the nurses before and during the first wave of the COVID-19 pandemic in Ali Ebn Abi Taleb hospital in southern Iran.

### Sample Size and Sampling

Sampling was performed before (from April to July 2019) and during the first wave of the COVID-19 pandemic (from April to July 2020), one year apart. Before the COVID-19 outbreak, 400 nurses were employed in Ali Ebn Abi Taleb hospital, while during the first wave of the COVID-19 pandemic, 500 nurses were employed in Ali Ebn Abi Taleb hospital. This hospital was the only COVID-19 referral hospital in Rafsanjan city, south-east Iran. In both cases, sampling was performed using the census method. Inclusion criteria were as follows: (1) nurses taking care of the patients, (2) nurses with one year of work experience, and (3) nurses who had spent at least 3 months at the hospital. Nurses with a history of mental disorders (self-reported) and incomplete questionnaires were excluded.

Before the COVID-19 outbreak, 279 frontline nurses out of 400 nurses completed the questionnaires, with 13 being excluded from the study because of high missing value. Therefore, the effective response rate 66.5% (*n* = 266) before the COVID-19 epidemic. During the first wave of the COVID-19 Pandemic, questionnaires were completed by 255 frontline nurses out of 500 nurses, 13 of whom were excluded from the study because of high missing value and one was excluded due to the history of mental disorders. Therefore, the effective response rate of the nurses was 48.4% (*n* = 242) during the first wave of the COVID-19 Pandemic. Power analysis calculations with G^∗^Power software (version 3.1.9.2) indicated that (power = 90% and *P* = 0.05) 235 participants would be needed in each group to detect an effect size of 0.3. Finally, 508 nurses’ data were used in the final analysis.

### Measurements

A three-part questionnaire was used for data gathering. A: Demographic information questionnaire B: Maslach Burnout Questionnaire, and C: Depression, Anxiety and Stress Scale (DASS-21).

### Demographic Information Questionnaire

The first section included of questions on demographic variables (i.e., gender, age, marital status, educational level, income, type of employment, work experience, ward, shift, and working hours per month).

### Maslach Burnout Inventory

The most common tool for burnout measurement is maslach burnout inventory (MBI). It includes 22 items and consists of three subscales, including emotional exhaustion (9 items), depersonalization (5 items) and reduced personal accomplishment (8 items). MBI is rated on a scale of 0 (never) to 6 (every day), with scores ranging from 0 to 54 for emotional exhaustion, 0 to 30 for depersonalization, and 0 to 48 for lack of personal accomplishment. High scores in emotional exhaustion, depersonalization and reduced personal accomplishment are considered as high burnout. The scoring procedure of MBI is as follows: emotional exhaustion: high (>26), medium (17–26), and low (<17); depersonalization: high (>12), medium (7–12), and low (<7); reduced personal accomplishment: high (>39), medium (32–39), and low (<32) (Kamali et al., 2020). In addition, Iranian researchers reported Cronbach’s alpha greater than 0.70 for 3 dimensions in nurses (Moalemi et al., 2018). In the present study, the Cronbach’s alpha coefficients of emotional exhaustion, depersonalization, reduced personal accomplishment, and the whole scale were 0.88, 0.72, 0.66, and 0.86, respectively.

### Depression, Anxiety, Stress Scale

Depression, anxiety, stress scale was developed by Lovibond in 1995 to assess three subscales of depression, anxiety and stress (Lovibond and Lovibond, 1995). Each scale of DASS-21 consists of seven items on a four-point Likert scale (never / low / medium / high). The lowest score is zero and the highest score is three, with the sum of the scores obtained being the final score of DASS-21. The final score of the subscales should be doubled. In Iran, Samani and Jokar reported the retest validity to be 0.80, 0.76, and 0.77 for depression, anxiety and stress, respectively. Cronbach’s alpha coefficients were reported to be 0.81, 0.74, and 0.78, for depression, anxiety and stress, respectively (Zakeri et al., 2021b). In the present study, the Cronbach’s alpha coefficients of depression, anxiety, stress and the whole scale were 0.88, 0.85, 0.87, and 0.94, respectively.

### Data Collection and Data Analysis

After obtaining the necessary permissions, the researcher referred to the research settings and started sampling in two hospitals in Rafsanjan. We collected data of 400 nurses from April to July 2019 before the COVID-19 outbreak and 500 nurses from April to July 2020 during the first wave of the COVID-19 pandemic. Thus, demographic information, MBI and DASS-21 questionnaires were distributed among the eligible nurses both before and during the first wave of the COVID-19 pandemic, and they completed the questionnaires in the presence of the researcher.

Descriptive statistics (frequency, percentage, mean and standard deviation) were used to describe demographic characteristics and mean scores of the questionnaires. Independent *t*-test, ANOVA test and multivariate linear regression test were used to determine the correlates of burnout, depression, anxiety, and stress scores before and during the first wave of the COVID-19 pandemic. A significance level of 0.05 was considered.

### Ethical Considerations

Ethical approval was obtained from the Ethics Committee of Rafsanjan University of Medical Sciences (IR.RUMS.REC.1397.099 and IR.RUMS.REC.1399.135). All participants received written information from the researcher and signed an informed consent form before inclusion in the study. The objectives of the study, the confidentiality and anonymity of the information were explained and participants were free to complete the questionnaire.

## Results

The mean age of the participants was 33.32 ± 6.12 and 33.07 ± 6.90 before and during the first wave of the COVID-19 pandemic, respectively. The demographic data of the participants before and during COVID-19 is presented in Table 1.

**TABLE 1:** Comparison of the demographic characteristics of the participants before and during the first wave of the COVID-19 Pandemic.

**Variables**	**Before COVID-19 (*n* = 266)**					**During the first wave of the COVID-19 pandemic (*n* = 242)**				
	***n* (%)**	**Burnout**	**Anxiety**	**Stress**	**Depression**	***n* (%)**	**Burnout**	**Anxiety**	**Stress**	**Depression**
**Gender**										
Male	50 (18.8)	*t* = 1.85 (0.06)	*t* = 0.62 (0.53)	*t* = −0.27 (0.78)	*t* = 0.50 (0.61)	68 (28.1)	*t* = 1.30 (0.23)	*t* = −0.90 (0.36)	*t* = −1.33 (0.18)	*t* = −0.43 (0.66)
Female	216 (81.2)					174 (71.9)				
**Marital status**										
Unmarried / widowed / divorce	47 (17.7)	*t* = −0.42 (0.70)	*t* = −0.97 (0.33)	*t* = −0.40 (0.68)	*t* = −1.05 (0.29)	59 (24.4)	*t* = 1.04 (0.29)	*t* = 1.25 (0.21)	*t* = 0.76 (0.44)	*t* = 0.80 (0.42)
Married	219 (82.3)					183 (75.6)				
**Number of children**										
0	106 (39.8)					79 (32.6)				
1	60 (22.6)	*F* = 0.86 (0.46)	*F* = 0.53 (0.66)	*F* = 0.80 (0.49)	*F* = 0.76 (0.51)	55 (22.7)	*F* = 1.08 (0.35)	*F* = 0.30 (0.82)	*F* = 0.79 (0.50)	*F* = 0.56 (0.63)
2	81 (30.5)					81 (33.5)				
3≤	19 (7.1)					27 (11.2)				
**Educational level**										
Bachelor	237 (89.1)	*t* = 0.19 (0.84)	*t* = −0.87 (0.38)	*t* = −0.62 (0.53)	*t* = −0.90 (0.36)	223 (92.1)	*t* = −0.80 (0.42)	*t* = −0.49 (0.62)	*t* = −0.55 (0.57)	*t* = 0.46 (0.64)
Masters	29 (10.9)					19 (7.9)				
**Income (million riyal)**										
<3	106 (39.8)	*F* = 0.86 (0.42)	*F* = 1.35 (0.26)	*F* = 1.34 (0.26)	*F* = 0.33 (0.71)	28 (11.6)	*F* = 2.85 (0.05)	*F* = 0.12 (0.88)	*F* = 0.76 (0.46)	*F* = 1.51 (0.22)
3–5	141 (53.1)					177 (73.1)				
>5	19 (7.1)					36 (15.3)				
**Type of employment**										
Hired	164 (61.7)	*t* = −0.69 (0.48)	*t* = 0.71 (0.47)	*t* = 1.09 (0.27)	*t* = 1.05 (0.29)	161 (66.5)	*t* = 0.46 (0.64)	*t* = −0.27 (0.78)	*t* = −0.64 (0.52)	*t* = −0.54 (0.58)
Contract recruiters^a^ / Committed^b^	102 (38.3)					81 (33.5)				
**Work experience (yr.)**										
>5	73 (27.4)					87 (36.0)				
5–10	120 (45.1)	*F* = 0.65 (0.58)	*F* = 2.73 (0.04)	*F* = 4.57 (0.004)	*F* = 4.16 (0.007)	67 (27.7)	*F* = 0.32 (0.81)	*F* = 0.79 (0.49)	*F* = 1.79 (0.14)	*F* = 1.12 (0.33)
11–15	40 (15.0)					38 (15.7)				
>15	33 (12.4)					50 (20.7)				
**Ward**										
Critical/intensive	76 (28.6)					89 (36.8)				
Emergency	44 (16.5)	*F* = 5.35 (0.001)	*F* = 1.77 (0.15)	*F* = 1.06 (0.36)	*F* = 2.00 (0.11)	65 (26.9)	*F* = 0.97 (0.40)	*F* = 0.54 (0.65)	*F* = 0.39 (0.75)	*F* = 0.55 (0.64)
Medical	90 (33.8)					59 (24.4)				
Others	56 (21.1)					29 (12.0)				
**Shift**										
Fixed	26 (9.8)	*t* = −2.16 (0.03)	*t* = −1.42 (0.15)	*t* = −1.17 (0.24)	*t* = −1.61 (0.10)	23 (9.5)	*t* = −0.13 (0.89)	*t* = −0.02 (0.98)	*t* = 1.00 (0.31)	*t* = 0.50 (0.61)
Rotational	240 (90.2)					219 (90.5)				
**Working hours (h) per month**										
<150	46 (17.3)					46 (19.0)				
150–160	100 (37.6)	*F* = 2.08 (0.10)	*F* = 2.70 (0.04)	*F* = 2.94 (0.03)	*F* = 2.57 (0.05)	108 (44.6)	*F* = 0.84 (0.47)	*F* = 0.87 (0.45)	*F* = 0.27 (0.84)	*F* = 0.65 (0.57)
161–170	80 (30.1)					49 (20.2)				
>170	40 (15.0)					39 (16.1)				

*Data were presented as number (%). *t*, independent *t*-test; *F*, analysis of variance test; ^*a*^, annually contracted with payment similar to hired nurses, ^*b*^, it is obligatory to work for government for 2 years at a lower rate of pay.*

**FIGURE 1:**
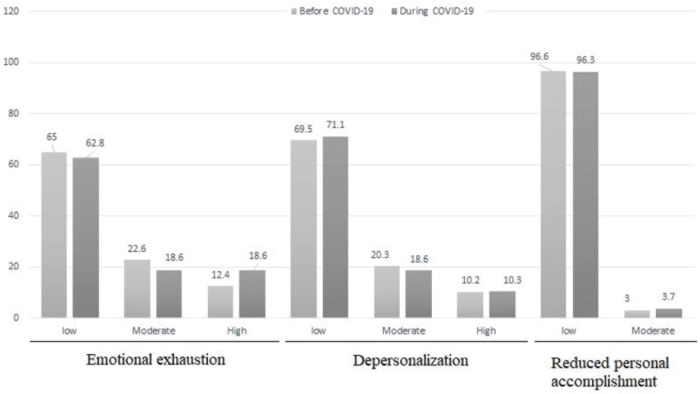
The comparison of the level of burnout score before and during the first wave of the COVID-19 pandemic.

The mean scores of burnout were 41.19 ± 17.21 and 43.25 ± 16.13 before and during the first wave of the COVID-19 pandemic, respectively. The scores of burnout and all its dimensions did not change significantly before and during the first wave of the COVID-19 pandemic (*p* > 0.05) (Table 2). Based on the findings, before the COVID-19 epidemic, 12.4% of nurse had high emotional exhaustion, 10.2% had high depersonalization, and 0.4% had reduced personal accomplishment. during the first wave of the COVID-19 pandemic, 18.6% of nurse had high emotional exhaustion and 10.3% had high depersonalization (Figure 1).

**TABLE 2:** Comparison of the burnout scores before and during the first wave of the COVID-19 pandemic.

**Group Variables**	**Before COVID-19 (*n* = 266)**			**During the first wave of the COVID-19 pandemic (*n* = 242)**			**Independent *t*-test**	***P*-value**
	**Median**	**Mean**	**Standard deviation**	**Median**	**Mean**	**Standard deviation**		
Emotional exhaustion	15.00	15.20	10.27	13.00	16.15	10.49	−1.02	0.30
Depersonalization	5.00	5.53	4.52	4.50	5.77	4.91	−0.56	0.57
Reduced personal accomplishment	21.00	20.45	6.72	21.00	21.32	5.77	−1.56	0.11
Burnout	38.00	41.19	17.21	41.00	43.25	16.13	−1.38	0.16

**FIGURE 2:**
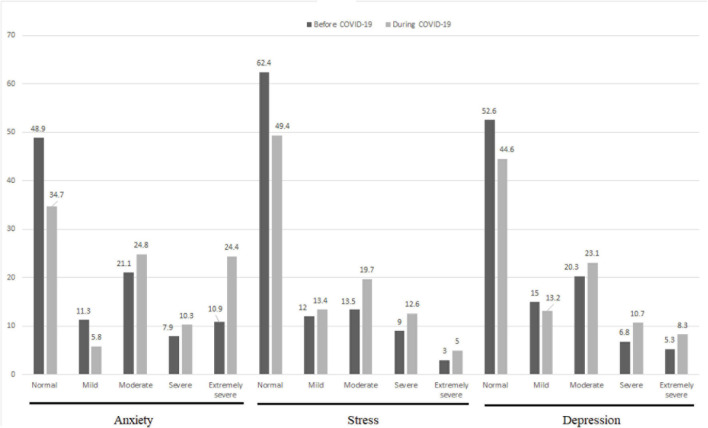
The comparison of the level of anxiety, stress, and depression score before COVID-19 and during the first wave of the COVID-19 pandemic.

Before the COVID-19 outbreak, the mean scores of anxiety, stress and depression were 8.74 ± 7.77, 13.71 ± 8.89 and 9.90 ± 8.41, respectively. during the first wave of the COVID-19 pandemic, the mean scores of anxiety, stress and depression were 12.65 ± 9.52, 16.23 ± 9.25, and 12.23 ± 9.25, respectively. Anxiety, stress and depression scores significantly increased during the first wave of the COVID-19 pandemic compared with before the COVID-19 outbreak (*p* < 0.05) (Table 3). The majority of the participants had normal level of anxiety, stress and depression before and during the first wave of the COVID-19 pandemic (Figure 2).

**TABLE 3:** Comparison of the anxiety, stress, and depression scores among nurses before and during the first wave of the COVID-19 pandemic.

**Group Variables**	**Before COVID-19 (*n* = 266)**			**During the first wave of the COVID-19 pandemic (*n* = 242)**			**Statistical test**	**Effect size**	***P*-value**
	**Median**	**Mean**	**Standard deviation**	**Median**	**Mean**	**Standard deviation**			
Anxiety	8.00	8.74	7.77	12.00	12.65	9.52	*Z* = −4.73	0.45	<0.001
Stress	12.00	13.71	8.89	16.00	16.23	9.25	*t* = −3.12	0.28	0.002
Depression	8.00	9.90	8.41	10.00	12.23	9.25	*Z* = −2.85	0.26	0.004

**t*, independent *t*-test; *Z*, Mann–Whitney U test.*

Before the COVID-19 epidemic, burnout had a significant association with types of ward and shifts. In addition, anxiety, stress, and depression had significant association with work experience and monthly working hours. However, during the first wave of the COVID-19 pandemic, none of the background information had significant association with burnout, anxiety, stress, and depression (Table 1).

## Discussion

The researchers wanted to see how occupational burnout, anxiety, stress, and depression affected nurses before and during the first wave of the COVID-19 pandemic. According to the results of this study, there was no significant difference in occupational burnout during and before the first wave of the COVID-19 pandemic. When compared with before the COVID-19, anxiety, stress, and depression scores increased significantly.

Barello et al. (2020) showed that occupational burnout of the healthcare workers was high during the COVID-19 epidemics (Barello et al., 2020). Guixia and Hui (2020) showed that the rate of occupational burnout of nurses was high during the COVID-19 outbreak (Guixia and Hui, 2020). Jalili et al. (2021) showed that burnout is prevalent among healthcare workers caring for COVID-19 patients. Age, gender, job category, and site of practice contribute to the level of burnout that the staff experience (Jalili et al., 2021). The results of Barello et al. (2020), Guixia and Hui (2020) and Jalili et al. (2021) were inconsistent with the results of present study. The current study compared the rate of occupational burnout before and during the first wave of the COVID-19 pandemic, whereas the previous studies measured occupational burnout during the first wave of the COVID-19 pandemic. Wu et al. (2020) showed that health care workers (physicians and nurses) working in the frontline wards of COVID-19 had lower occupational burnout rates than health care workers working in non-COVID-19 wards (Wu et al., 2020). The present study compared nurses’ occupational burnout before and during the first wave of the COVID-19 pandemic, but Wu et al. compared occupational burnout between healthcare workers in non-COVID-19 wards and frontline wards.

According to Gómez-Urquiza et al. (2017), burnout syndrome is very common among emergency nurses (Gómez-Urquiza et al., 2017). According to Zhang et al. (2014), intensive care unit nurses have a high level of occupational burnout (Zhang et al., 2014). The current study compared nurses working in all wards (intensive care, emergency, medical, and so on) at two different times (before and during the first wave of the COVID-19 pandemic), but Zhang only studied intensive care unit nurses, while Gómez-Urquiza studied emergency nurses, and no comparison was made in both studies.

Zakeri et al. (2021a) and Zheng et al. (2021) showed the high prevalence of depression and anxiety among nurses during the COVID-19 pandemic (Zakeri et al., 2021a; Zheng et al., 2021). Doo et al. (2021) revealed that nurses working in the COVID-19 wards had higher anxiety and depression than nurses working in other wards (Doo et al., 2021). Tiete et al. (2020) showed that healthcare workers (nurses and physicians) working in the COVID-19 wards had higher levels of anxiety, stress, and depression than health care workers working in other wards (non-COVID-19 wards) (Tiete et al., 2020). These studies are consistent with the present study because COVID-19 epidemic affects nurses’ mental health and increases their anxiety, stress and depression.

Previous studies on the prevalence of Severe Acute Respiratory Syndrome (SARS) and Middle East Respiratory Syndrome (MERS) have shown that healthcare workers are not only under stress during an outbreak, but may also be psychologically affected long after the initial outbreak (Lee et al., 2005; Khalid et al., 2016). Alizadeh et al. showed that there were some barriers and challenges to medical personnel exposed to COVID-19 that caused psychological distress. Some of these problems are related to the nature of illness, others are related to social and organizational demands, and some supportive resources buffer the relationship between occupational demands and psychological distress (Alizadeh et al., 2020). Despite the fact that each epidemic differs significantly in terms of geographical location, pathogenic characteristics, transmission route, infection, mortality, and treatment availability, previous studies have found that epidemics have a significant impact on the psychological state of healthcare workers (Jiang, 2020).

During the COVID-19 epidemic, healthcare workers play an important role in the treatment and care of the patients with COVID-19. Healthcare workers are under a lot of stress during the epidemic (high risk of infection, concern about patients’ treatment, etc.) (Liu et al., 2021). Physical, mental, and physical disorders result from the problems they experience at workplace, which lead to occupational burnout, anxiety, stress and depression (Wan et al., 2020). Occupational burnout is defined as a state of chronic work-related stress that reduces job satisfaction and can have a negative impact on nurses’ efficiency, occupational advancement, and work quality (Wang et al., 2019). Anxiety causes a person not to use his/her abilities and talents properly. Job stress has a significant impact on physical and mental illness. High levels of stress can impair healthcare workers’ performance as well as negatively affect their attitudes and behaviors (Vilagut et al., 2016). Depression is one of the five debilitating diseases and is predicted to be one of the major challenges in developed countries by 2030 (Zhang et al., 2020). Physical and mental health of nurses is important to provide health care during the COVID-19 epidemic. It is essential to diagnose, control and treat these disorders as soon as possible. Given the high prevalence of stress, anxiety and depression in healthcare workers caring for the COVID-19 patients (Zakeri et al., 2021a, c), managers should pay more attention to the symptoms of these disorders and take steps to reduce them. Healthcare workers can be protected from anxiety, stress, depression, and occupational burnout by taking steps like seeking help from mental health professionals, getting enough rest, eating well, exercising regularly, and resting when they are tired (Guixia and Hui, 2020).

## Limitations

There were some limitations to this study. Since the current study was conducted early in the COVID-19 epidemic, the long-term effects of burnout, anxiety, stress, and depression are dependent on the extent of COVID-19 prevalence. These factors may be influenced by current efforts to adapt the workplace to new conditions (such as providing protective equipment or increasing the number of health care professionals). As a result, a future follow-up study over several months is required. Another limitation is the cross-sectional design of the study. Because the epidemic is still ongoing, we are unable to demonstrate its impact on mental health in this study.

## Conclusion

The current study found that the COVID-19 epidemic had an impact on the frontline nurses’ mental health and increased their anxiety, stress, and depression. Occupational burnout was not different before and during the first wave of the COVID-19 pandemic. Physical and mental health of nurses is important to provide health care during the COVID-19 epidemic. Therefore, health care authorities and decision makers, at the national and international levels, should take measures to reduce these disorders in nurses who are in direct contact with COVID-19 patients, which increase the productivity of hospital staff, speed up epidemic control, and provide effective treatment for the COVID-19 patients.

## Data Availability Statement

The raw data supporting the conclusions of this article will be made available by the authors, without undue reservation.

## Ethics Statement

The studies involving human participants were reviewed and approved by Ethics Committee of Rafsanjan University of Medical Sciences (IR.RUMS.REC.1397.099 and IR.RUMS.REC.1399.135). The patients/participants provided their written informed consent to participate in this study.

## Author Contributions

MZ and MD: conceptualization, supervision, methodology, data analysis, and writing – reviewing and editing. ER, FS, and HG: conceptualization, data curation, software, and writing – original draft preparation. All authors contributed to the article and approved the submitted version.

## Conflict of Interest

The authors declare that the research was conducted in the absence of any commercial or financial relationships that could be construed as a potential conflict of interest.

## Publisher’s Note

All claims expressed in this article are solely those of the authors and do not necessarily represent those of their affiliated organizations, or those of the publisher, the editors and the reviewers. Any product that may be evaluated in this article, or claim that may be made by its manufacturer, is not guaranteed or endorsed by the publisher.
